# Variations in Macular Pigment Optical Density in Children and Adolescents Depending on Time Spent on Smartphones

**DOI:** 10.3390/vision10020030

**Published:** 2026-05-15

**Authors:** Livia Hopîrcă, Alexandru Țîpcu, Mădălina-Claudia Hapca, Iulia-Andrada Nemeș-Drăgan, Cosmina Teodora Lazăr, Simona Delia Nicoară

**Affiliations:** 1 Doctoral School, “Iuliu Hațieganu” University of Medicine and Pharmacy, 400012 Cluj-Napoca, Romania; alextipcu@gmail.com (A.Ț.); simonanicoara1@gmail.com (S.D.N.); 2 Department of Ophthalmology, “Dr. George Trifon” Hospital, 425200 Năsăud, Romania; 3 Department of Radiotherapy, Oncohelp Center of Oncology, 300239 Timișoara, Romania; 4 Ophthalmology Department, “Iuliu Hațieganu” University of Medicine and Pharmacy, 400012 Cluj-Napoca, Romania; madalina.prodan06@gmail.com (M.-C.H.); d_iulia_a@yahoo.com (I.-A.N.-D.); 5 Ophthalmology Department, Emergency County Hospital, 400006 Cluj-Napoca, Romania; 6 Surgery Department, Medicine and Pharmacy Faculty, University of Oradea, 410073 Oradea, Romania; cosvic30@yahoo.com; 7 Neonatology Department, Emergency County Hospital, 410169 Oradea, Romania

**Keywords:** children, smartphone, lutein, macular pigment, blue light, screen time, macular pigment optical density

## Abstract

Background: Children and teenagers use electronic devices daily, especially smartphones. The use of these devices exposes children and adolescents to excess blue light, which can alter the structures of the eye, especially the retina. As a protective mechanism, the macular region contains pigments represented by lutein, zeaxanthin, and meso-zeaxanthin. In this study, we aimed to analyze the relationship between the Macular Pigment Optical Density (MPOD) levels in the macula and the time spent on smartphones in children and adolescents. Methods: Fifty-seven children and teenagers aged between 8 and 18 were evaluated, with a total of 114 eyes. The patients included in the study were divided into two groups: those who spent less than two hours a day on the device and those who exceeded this period. To determine the amount of macular pigment, the Heterochromatic Flicker Photometry technique was used. Results: We found a statistically significant difference in screen time between weekdays and weekends in favor of the latter. We compared the different refractive categories with respect to pigment levels and screen time and found no significant differences between groups. When comparing the patients with respect to environment, we found a slight difference in macular pigmentation in the favor of rural areas and also in the screen time which was shorter in rural areas. We found a strong association at all levels between longer screen time (both weekdays and weekend) and lower macular pigment quantities for both eyes. When comparing the groups with more/less than 2 h of screen time, the MPOD was lower for both eyes in the group with over 2 h screen time. Conclusions: In this study we demonstrated that smartphone use is a risk factor leading to a decrease in MPOD in children and adolescents. The amount of lutein in the retina, brain and serum are correlated, therefore MPOD can be considered a natural biomarker of lutein and zeaxanthin levels in the body.

## 1. Introduction

Children and adolescents are constantly exposed to electronic devices, especially smartphones. This leads to a considerable increase in the rate of screen addiction, which represents a global problem [1]. Mobile phones have important functions, from internet access to social networking, telecommunications, email, calendars, instant access to encyclopedias and books in all fields. Also, educational opportunities, social connections and access to health information may be obtained [2]. Smartphones give us total control and are a tool that children and adolescents become addicted to. These devices shape their lives, with most preferring a sedentary lifestyle in which Virtual space gains ground at the expense of time spent in nature or with real friends. Adverse reactions are inevitable [3].

For children over 8 years of age and adolescents, guidelines recommend using devices for a maximum of 2 h per day. These 2 h are considered a safe zone, within which children’s development would not be affected. However, in reality, this period is longer, with consequences that could be unfavorable [4].

Excessive use of devices has various side effects, such as decreased school performance, interest in studying, motivation, and sleep disorders, but also obesity and musculoskeletal problems like back and neck pain [5,6]. Device addiction also leads to school burnout, disinterest, and negative psychological reactions [7], behavioral disorders, various emotions, generalized anxiety, and health problems, even from an early age. High blood pressure and metabolic disorders have been described, conditions that are on the rise in the pediatric population and are considered to be related to the sedentary lifestyle of young people addicted to screens [8,9].

Computer vision syndrome has been described as a combination of symptoms that arise as a result of excessive exposure to digital devices. This is characterized by a range of ocular symptoms, including eye strain, itching and burning sensations, red eyes, blurred vision, and dry eyes. This syndrome also presents extraocular symptoms, such as neck, shoulder, and back pain due to poor posture. Using these devices for more than two hours significantly increases the risk of developing Computer Vision Syndrome [10].

In this process, one of the target organs, without which the use of digital screens could not take place, is the eye. The widespread use of LED-backlit screens has changed the way people read, as the light illuminates the text directly on screens, rather than reflects it off the text on paper. This increases the human eye’s exposure to blue light [11]. Blue light (wavelength between 380 and 500 nm) has the highest energy in the visible light spectrum [12] and penetrates ocular tissues, being able to reach the retina with relatively little loss of harmful potential [13]. The main destructive mechanisms are oxidative stress, mitochondrial apoptosis and DNA damage. The retina and ocular surface are vulnerable to this danger [14]. The oxygen demand of photoreceptor cells is 3–4 times higher than that of other neurons in the retina and central nervous system. The estimates of the oxidative metabolism rate in photoreceptor cells are among the highest in the human body [13].

As a protective mechanism, in the retina there are macular pigments with maximum concentrations in the fovea, represented by lutein (L), zeaxanthin (Z), and meso-zeaxanthin (MZ) [15]. Macular pigments cannot be synthesized by the body, which is why they must be obtained through a balanced diet. Nutrition plays a vital role in human growth, development, and health, especially among children and adolescents. The eyes require special attention in this regard, as they are the organs responsible for processing images from devices. Foods that contain high concentrations of L and Z include green leafy vegetables, especially kale, broccoli, spinach, peas, lettuce, parsley, and oregano, as well as brightly colored vegetables such as red peppers. Significant concentrations are also found in egg yolks and grains like corn and certain types of wheat [16].

Pigments give the macula a yellowish color, which is why this region is called Macula Lutea or the yellow spot. This area has a diameter of approximately 2 mm and contains most of the cone photoreceptor cells (responsible for daylight, fine and color vision) [17]. The human body cannot synthesize L and Z; therefore, these pigments must be obtained from food. MZ is synthesized in the macula through the conversion of L [18]. L is the dominant carotenoid in the periphery of the macula, Z predominates in the mid periphery, and MZ has its maximum concentration in the fovea [15]. They act as antioxidants and light filters and play a role in light absorption, especially UV, which peaks at 460 nm. The three pigments are distributed differently in the retina, with distinct light absorption patterns. Although they have structural differences and absorption spectra, these pigments (MZ, L, and Z) lead to optimal collective filtering of blue light at the macula, which would not be achieved by any of these carotenoids alone [19]. In the fovea, the concentration of carotenoids is close to 1 mM, and the ratio of L, Z and MZ is 1:1:1. The concentration of macular carotenoids decreases more than 100-fold just a few millimeters away from the foveal center, and the composition ratio approaches 3:1:0 in the peripheral retina, meaning that the concentration of L is considerably higher in this area [20]. L is not only the most important carotenoid in the retina [21], but it also fulfills other roles in the body, such as preventing cognitive decline [22], enhancing memory, attention, and brain function, protecting the vessels in diabetic retinopathy and the skin from sun exposure [23].

Since the role of macular pigments in the pediatric population is uncertain [24], in this study, we aimed to analyze the impact of the time spent on screens on the amount of macular pigment in children and teenagers.

## 2. Materials and Methods

This is an observational study that aims to evaluate possible risk factors in the young population. Fifty-seven children and teenagers aged between 8 and 18 years were evaluated, totaling 114 eyes. This study was approved by the Ethics Committee of the “Iuliu Hatieganu” University of Medicine and Pharmacy from Cluj-Napoca, Romania and “Dr. George Trifon” Năsăud Hospital, Romania.

All the subjects, together with their parents or legal guardians, signed the informed consent form, which was explained to them in detail. They agreed that the data collected could be used for scientific purposes and that the results could be published. Data was collected only after signing the written informed consent form.

All children were examined by an ophthalmologist. The subjects underwent an ophthalmological examination, which consisted of measuring intraocular pressure, biomicroscopic examination of the anterior and posterior segments, measurement of visual acuity and contrast sensitivity. All subjects were evaluated for refractive status, and cycloplegia was performed to rule out possible diagnostic errors. They were also assessed in terms of their development, using body mass index (BMI), which is a measure of weight relative to height. For children and teens, BMI is interpreted using sex-specific BMI-for-age percentiles recommended by the World Health Organization.

Screen time was calculated based on an objective measure. Both the participants and their parents agreed to share their phone data showing their average daily screen time. To this aim, the data recorded by the smartphone was used.

Exclusion criteria from the study were: the presence of amblyopia or any other eye condition (such as strabismus, glaucoma, cataract, uveitis, keratitis or other ocular abnormalities), systemic diseases, the use of supplements and protective glasses while using screens, as well as lack of consent.

The patients included in the study were divided into two groups: those who spent less than two hours a day on the device and those who exceeded this period.

Since there are many eye conditions that progress asymmetrically, such as refractive errors in cases of anisometropia [25], keratoconus, glaucoma, optic neuropathies or retinal degenerative diseases [26], we felt it was important to examine each eye separately.

During the preparation of this article, no AI tool was used.

To determine the amount of macular pigment, the Heterochromatic Flicker Photometry (HFP) technique was used, a psychophysical method based on the Macular Pigment Screener MPS II (Elektron Technology UK Ltd., Cambridge, UK). This is a non-invasive, rapid method with good reproducibility (0.97). This method has proven to be reliable when used in children [27].

This measurement is accomplished by viewing a small circular stimulus that alternates between a test wavelength that is absorbed by the MP (typically—blue, 460 nm) and a reference wavelength that is not absorbed (typically—green, 540 nm). The patient is instructed to fixate on the central target, and then to press the button when observing a flicker in the target [28]. Flicker observed by the subject is reduced to a null point by adjusting the intensity of the former while viewing the stimulus centrally, and then peripherally [29]. This is a fast assessment that takes about 90 s per eye. Before the main test, there is a short familiarization test, lasting about 30 s, which helps the patient understand what the assessment entails and what they need to do. The examination of both eyes takes about 5 min. The MPOD measured is adjusted to account for the normal age-related yellowing of the lens and a final MPOD is reported in density units [28].

### Data Analysis

Nominal data were expressed in terms of frequencies and percentages and numerical data as mean ± standard deviation and/or median and IQR (Interquartile Range). Distribution analysis was performed using Kolmogorov–Smirnov and Shapiro–Wilk tests, alongside every derived distribution graph (histograms and Q-Q plots). Analysis for independence was performed using Chi-squared/exact Fisher testing. Independent groups were compared using either an independent *t*-test, ANOVA (3+ groups, Tukey HSD as post hoc), Mann–Whitney U or Kruskal–Wallis tests (3+ groups, Dunn-Bonferroni as post hoc), according to distribution models. Dependent groups were compared using either a paired *t*-test or a Wilcoxon signed-rank test, according to distribution. Linear relationships were assessed using Spearman correlations. All tests were 2-sided. The cutoff for declaring significance was set at an alpha (Pearson’s *p*) of 0.05. All database processing, data analysis, and chart generation were performed using IBM SPSS Statistics v.26.0.0.

## 3. Results

We analyzed a total of 57 participants. Patient characteristics are represented in Table 1.

**Figure 1:**
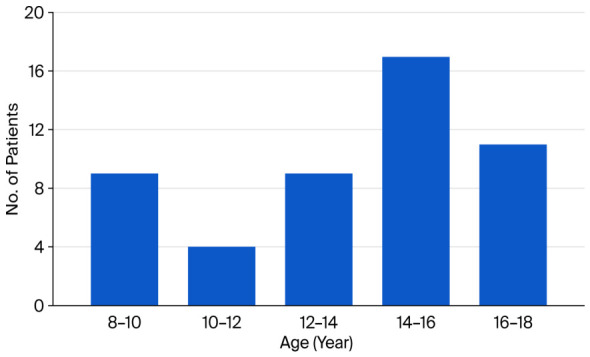
Age distribution histogram.

Figure 1 shows the overall age distribution of the study population, unstratified.

Table 2 presents a comparative analysis of Macular Pigment Optical Density (MPOD) across different age groups and weekly screen time exposure. As screen time increases, the MPOD significantly decreases across all age groups. The highest protection (0.77) is found in the 13–15 age group with minimal screen exposure (<1 h). While younger children start with higher values, the 16–18 age group shows a more rapid decline in pigment density when moving into the high-exposure categories (4–6 h).

**Figure 2:**
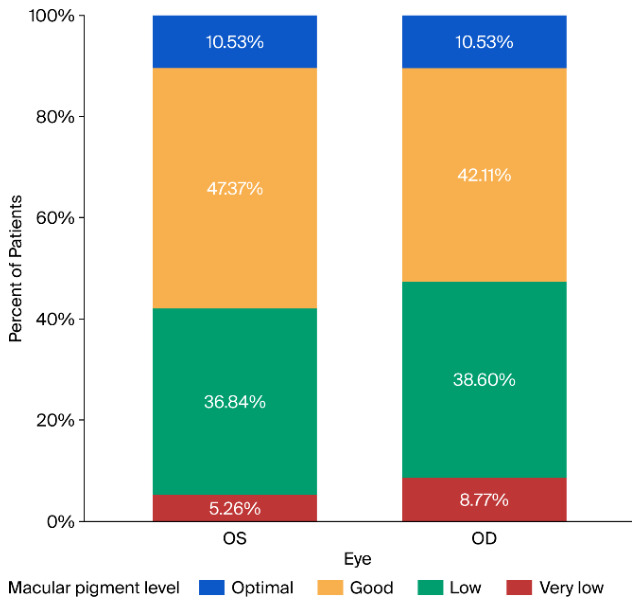
Macular pigment levels, separated for each eye.

Macular pigment levels (optimal >0.75, good between 0.5 and 0.75, low between 0.25 and 0.5 and very low <0.25) and differences (*p* = 0.440) are highlighted in Figure 2.

**Figure 3:**
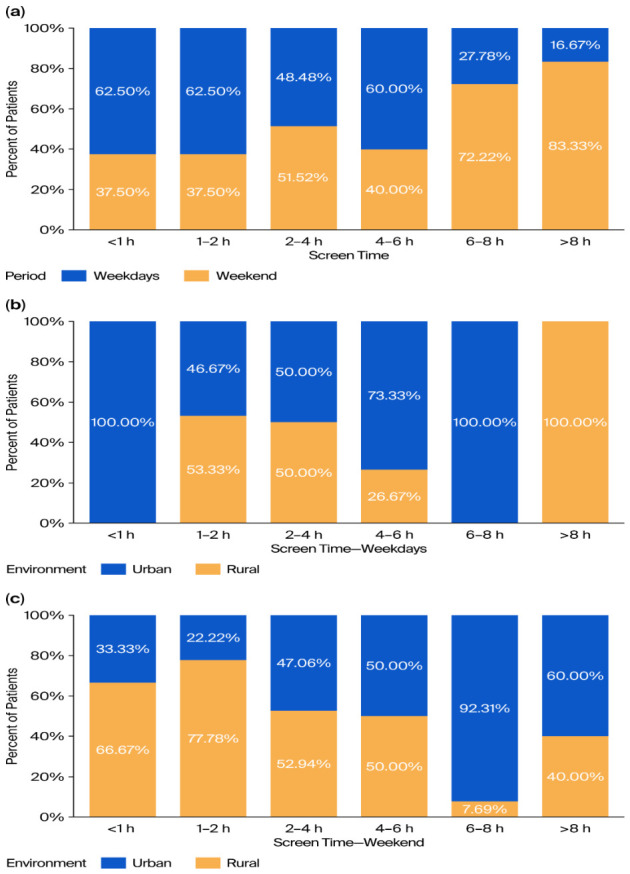
(**a**) Screen time categories, separated by period (weekdays vs. weekend), (**b**) screen time (weekdays) separated by environment, (**c**) screen time (weekends) separated by environment.

We found a statistically significant difference in screen time between the weekdays and weekend (*p* = 0.001), with a trend showing higher overall screen time during the weekend, highlighted in Figure 3a–c.

We compared the different refractive categories with respect to pigment levels and found no significant differences between groups (*p* = 0.789 for OS and *p* = 0.771 for OD). Likewise, no significant differences were found between these groups with respect to screen time (*p* = 0.899 for weekdays and *p* = 0.788 for weekends).

When comparing the patients with respect to environment, we found a slight difference in macular pigmentation (*p* = 0.071 for OS and *p* = 0.049 for OD) in favor of rural areas. In the urban area, OS had a pigment level of 0.487 ± 0.179, OD had a pigment level of 0.476 ± 0.175, whereas in the rural area, OS had a pigment level of 0.576 ± 0.186 and OD had a pigment level of 0.572 ± 0.184, highlighting an overall trend for a higher pigment level in the rural area. This observation is further supported by the differences we found in screen time between the two groups (*p* = 0.004 for weekdays and *p* = 0.004 for weekends). These latter differences are highlighted in Figure 2 and Figure 3.

**Figure 4:**
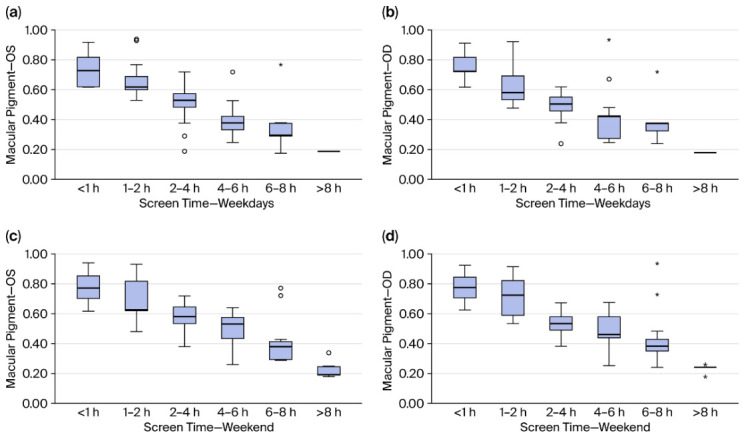
(**a**–**d**) Relationship between screen time and macular pigment. Circles indicating outlier case outside of 1.5xIQR. Star indicating outlier case outside of 3xIQR.

We found a strong association at all levels between screen time (both weekdays and weekends) and macular pigment quantities for both eyes. These are highlighted in Table 3 and Figure 4.

**Figure 5:**
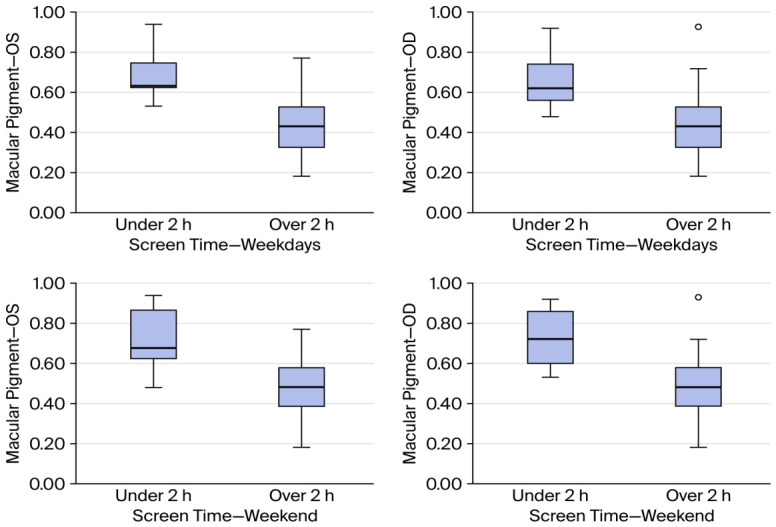
Relationship between screen time (2 h cut-off) and macular pigment. Circle indicating outlier case outside of 1.5xIQR.

Finally, we aimed at comparing the groups with more/less than 2 h of screen time on weekdays and the weekend. In terms of weekdays, the macular pigment density was lower for both eyes in the group with over 2 h screen time (0.442 ± 0.155 vs. 0.686 ± 0.127, *p* < 0.001 for OS and 0.440 ± 0.156 vs. 0.667 ± 0.137, *p* < 0.001 for OD). Similar results were found in the case of weekends (0.477 ± 0.160 vs. 0.716 ± 0.159, *p* < 0.001 for OS and 0.464 ± 0.152 vs. 0.728 ± 0.142, *p* < 0.001 for OD). Figure 5 illustrates these differences.

## 4. Discussion

The main purpose of the current study was to evaluate the relationship between the MPOD levels in macula lutea and the time spent on smartphones in children and adolescents.

This study confirmed that prolonged smartphone use poses a risk of changes in the amount of macular pigment. Furthermore, we found that daily use of the smartphone for more than two hours can lead to a significant decrease in the amount of macular pigment, with potential repercussions on the retinal structure and implicitly promote the early onset of degenerative eye diseases. However, extensive research is needed to confirm this finding.

Several studies have been conducted on adults, which have determined a decrease in MPOD in people who spend long periods of time in front of screens [30,31,32]. The blue light emitted by digital screens directly affects macular pigment levels [30].

Prolonged use of smartphones exposes the eyes to a considerable amount of blue light, which can influence performance levels, well-being [33] and the secretion of the hormone melatonin [34], which is involved in regulating the circadian rhythm. However, blue light also has positive effects, such as stimulating cognitive brain activity [35] and increasing physical performance [36].

With the ubiquity of screen-based technology and internet accessibility, there has been a dramatic increase in screen time among young adults, leading to a radical increase in the prevalence of various eye diseases that cause blindness. Prolonged exposure to relatively high-energy, short-wavelength light tends to cause a series of cumulative long-term changes, including irreversible cellular damage to photoreceptors and the retinal pigmented epithelium (RPE). However, little is known about their effects in young adults, especially in children exposed to digital screens, particularly smartphones, on a long-term and repeated basis [37].

There are studies showing the link between increased intake of L and Z and improved visual function, as measured by visual acuity, contrast sensitivity and glare [38,39,40,41]. Also, people with lower levels of L and Z have an increased risk of photophobia compared to those with high pigment levels who tolerate light much better [42].

The amount of lutein in the retina, brain and serum is correlated; therefore, MPOD can be considered a natural biomarker of lutein and zeaxanthin levels in the body [43], and its decrease in the macula could also be associated with its decrease in the brain [44]. The levels of these carotenoids in the retina and brain during the early stage of life play an important developmental role in visual and cognitive health [45]. Increased levels of L and Z have been associated with superior cognitive function. Studies have been conducted on children to analyze cognitive and visual function and carotenoid levels. The higher the carotenoid levels, the higher the cognitive and visual function, as well as the speed of visual information processing. The areas critical for information processing, represented by the hippocampus, frontal cortex, and occipital cortex, have high concentrations of lipid-soluble antioxidants, represented by L and Z [45]. Lutein has been consistently associated with superior cognitive performance, possibly due to its antioxidant and anti-inflammatory action, but also to increasing the stability of the neuronal membrane [46]. On the other hand, multiple studies confirm that spending a long time on the smartphone is associated with behavioral disorders and decreased cognitive performance [47].

Other studies that evaluated visual function in patients who spent a lot of time in front of screens (more than 6 h a day) concluded that L and Z play a very important protective role, and supplementing the body with these carotenoids significantly improves both visual and cognitive function [30,31].

Other ocular parameters were also evaluated in children who spent a lot of time in front of screens. Thus, an increased rate of eye fatigue, blurred vision, eye irritation, and burning sensation was described, the so-called computer vision syndrome, while patients who used their phones for less than 60 min/day reported fewer or no previously described symptoms [48].

Low macular pigment levels have been associated with a number of eye conditions, the most important of which is age-related macular degeneration (AMD). It is a chronic, degenerative, progressive disease with a tendency toward bilateral involvement, affecting the macula, the central area of the retina responsible for detailed vision. There is currently no cure for this retinal condition, as the damage is irreversible [49]. Therefore, macular pigments play an important role in physiological protection against AMD, and it has been hypothesized that lower MPOD could be a risk factor for the development of this disease. The decrease in L and Z in children who spend a lot of time on smartphones could be correlated with the early onset of AMD, but only time will tell, as these devices have been part of our lives for less than 30 years, and the time elapsed is insufficient to assess the possible negative effects. That is why we must take protective measures and use blue light-emitting devices wisely.

The link between screen time and the onset of retinal diseases in adults, particularly AMD, has been evaluated. The longer this time, the higher the incidence rate of these diseases, and the use of devices is considered a risk factor in the onset of AMD, but also in the decrease in macular thickness (certified on OCT). Reducing the time spent on devices could be a protective factor against retinal diseases [50].

Research is also being conducted on the link between lutein levels and myopia. Given that myopia is a condition with an increasing incidence among children, this link could play an important role in the onset and progression of the disease [51]. Myopia is a refractive error caused primarily by an increase in the axial length of the eyeball and it carries a risk of complications such as retinal detachment, subretinal neovascularization, early-onset cataracts, and glaucoma [52]. Low lutein levels have been correlated with decreased choroidal blood flow and choroidal thinning [53], which we know is a primary factor that predisposes the eye to myopic shift. Lutein supplementation has been shown to protect the choroid and maintain its normal thickness, thus protecting the eye from the myopic shift [51].

The link between screen time and myopia was also evaluated. The longer the screen time, the higher the rate of myopia. Ongoing studies are set to identify risk factors and methods for preventing this condition and it has been demonstrated that exposure to devices for less than one hour per day is associated with a lower risk of developing myopia [54].

On the other hand, some studies defend these devices (computers/smartphones), claiming that constant attention and interaction would reduce the risk of cognitive decline by stimulating thought processes, according to studies conducted on the elderly population [55].

As a protective measure, Bharadwaj VG et al. recommend supplementation with lutein and zeaxanthin for healthy individuals who spend more than 8 h a day in front of screens. This supplementation has been associated with an increase in MPOD, as well as improved visual function and retinal thickness [30].

Javier Vicente-Tejedor et al. found that the use of blue light blocking filters could significantly protect retinal function and morphology. He conducted an experiment in which a group of laboratory animals was exposed to a considerable amount of light (5000 lux) for 7 consecutive days. Animals protected by filters that blocked 94% of blue light had much better responses, with better preservation of retinal structure and higher photoreceptor survival compared to unprotected animals. On the other hand, unexposed animals had the best responses, both functionally and morphologically [56,57].

An important factor in protecting children from the virtual world is the relationship they have with their parents. Clear rules regarding the time of use, as well as parents’ discerning use of screens, are protective factors in terms of exposure to devices [58]. Parental guidelines provided by clinicians would be needed, clearly establishing the limits of screen exposure, as well as the quality of content, in addition to co-use strategies.

As a protection method, it is recommended to increase consumption of foods rich in carotenoids, as well as to take lutein supplements, and wear blue-light-filtering glasses while using electronic devices. Parents should also be more involved and establish clear rules regarding the use of digital devices and screen time.

Some Raman scattering studies suggest that MPOD tends to be lower in older individuals and that there is a decline of over 10% per decade [59,60]. In contrast, other studies have not identified age-related decline in MPOD [61,62]. Lutein levels may remain similar to those found in younger people, which is reasonable, given that they are closely correlated with diet and lifestyle.

Pupil size may affect the accuracy of MPOD measurements depending on the method used. The HFP method has proven effective and reproducible in children [63]. When using this method, pupil dilation is not necessary, whereas the Fundus reflectometry method, although more accurate and precise, needs a larger pupil for the measurement to be correct; however, the intensity of the blue light required for this measurement has proven too high for children [64]. Similarly, when using Fundus Autofluorescence, there is a difference in measurements depending on the state of the pupil [65], with pupil dilation being necessary for reliable results. If the pupil is not properly dilated, underestimations or inaccuracies may occur during testing. These measurements require expensive equipment, which also limits their widespread use.

This study revealed that subjects from rural areas have higher levels of macular pigment compared to those from urban areas. This may be influenced by several factors. One factor is exposure to electronic devices, which was found to be lower, according to the data from this study. However, the different lifestyles between the two environments may also contribute to this. In rural areas, children spend more time outdoors, exposed to more sunlight. This has been shown to be a factor that leads to increased MPOD [66]. Additionally, the diet in this environment may be of superior nutritional quality [66].

There are other factors that lead to a decrease in MPOD levels, among which smoking, along with an unbalanced diet, are the main ones [67,68,69]. Both vegetarian and obese patients have low levels of L and Z. The explanation lies in the fact that these pigments require protein and cholesterol to be absorbed in the intestinal tract [70] and, since they are fat-soluble, are stored in fat cells. As a result, there is competition between the retina and adipose tissue for the uptake of L and Z [67,71]. Additionally, certain kidney diseases lead to a decrease in these pigments through their excessive excretion [72].

In contrast, increased exposure to sunlight and a balanced diet would raise carotenoid limitation levels [66].

Our study has some limitations. Firstly, it was conducted on a relatively small number of participants. Although this sample size is sufficient for an observational study, it limits the generalizability of the results to the broader pediatric population, which is why further studies involving a larger number of participants who undergo objective measurements of MPOD levels, like dual-wavelength autofluorescence, are needed.

Secondly, the participants included in this study were assessed regarding their use of dietary supplements and daily consumption of fruits and vegetables, but a food frequency questionnaire (FFQ) was not used. This is a limitation of the study, which will be addressed by including an FFQ in future research.

Thirdly, McCorkle et al. (2015) showed that HFP, even if far more readily available for measuring MPOD, including in preadolescent children [27], demonstrates only moderate reliability and depends on subjective responses. This introduces variability related to attention and task comprehension, which can increase noise in macular pigment measurements in children. Therefore, in future studies, we will consider better testing methods which provide objective measurements.

## 5. Conclusions

In this study, we demonstrated that smartphone use is a risk factor leading to a decrease in MPOD in children, which is linear, meaning that the longer the time spent, the greater the decrease in pigment density. According to our knowledge, no similar studies have been conducted on children to date. Reducing the time spent on smartphones seems reasonable in order to maintain an optimal level of macular pigment. Using smartphones for less than 2 h a day in children and adolescents has been shown to be effective in maintaining optimal macular pigment levels. Future research should identify the effects of MPOD on retinal function and structure, as well as the influence it has on brain health and information processing.

## Figures and Tables

**Table 1:** Patient characteristics, stratified by gender.

Variable		Male (21)	Female (36)	Both Genders (57)	*p*-Value
Age		14 (IQR 7)	14 (IQR 4)	14 (IQR 4)	0.828
Environment	Urban	13 (22.8)	18 (31.6)	31 (54.4)	0.384
	Rural	8 (14)	18 (31.6)	26 (45.6)	
Refractive category	Emmetropia	18 (31.6)	28 (49.1)	46 (80.7)	0.242
	Myopia	0 (0)	5 (8.8)	5 (8.8)	
	Hypermetropia	1 (1.8)	2 (3.5)	3 (5.3)	
	Astigmatism	2 (3.5)	1 (1.8)	3 (5.3)	
Macular pigment—OS *		0.501 ± 0.180	0.543 ± 0.190	0.527 ± 0.186	0.410
Macular pigment—OD *		0.481 ± 0.161	0.542 ± 0.195	0.520 ± 0.184	0.238
Screen time (weekdays)	Under 1 h	1 (1.8)	4 (7)	5 (8.8)	0.932
	1–2 h	5 (8.8)	10 (17.5)	15 (26.3)	
	2–4 h	8 (14)	8 (14)	16 (28.1)	
	4–6 h	6 (10.5)	9 (15.8)	15 (26.3)	
	6–8 h	1 (1.8)	4 (7)	5 (8.8)	
	Over 8 h	0 (0)	1 (1.8)	1 (1.8)	
Screen time (weekend)	Under 1 h	1 (1.8)	2 (3.5)	3 (5.3)	0.435
	1–2 h	3 (5.3)	6 (10.5)	9 (15.8)	
	2–4 h	5 (8.8)	12 (21.1)	17 (29.8)	
	4–6 h	4 (7)	6 (10.5)	10 (17.5)	
	6–8 h	6 (10.5)	7 (12.3)	13 (22.8)	
	Over 8 h	2 (3.5)	3 (5.3)	5 (8.8)	

* OS—oculus sinister; OD—oculus dexter.

**Table 2:** MPOD distribution by age group and weekly screen time.

Screen Time (Weekly)	9–12 Years	13–15 Years	16–18 Years
<1 h	0.76	0.77	0.67
1–2 h	0.65	0.71	0.55
2–4 h	0.34	0.51	0.57
4–6 h	0.32	0.45	0.34
6–8 h	0.32	-	0.44
>8 h	-	0.18	0.14

**Table 3:** Spearman analysis between screen time and macular pigment levels.

		Macular Pigment—OS	Macular Pigment—OD	Screen Time— Weekdays	Screen Time— Weekend
Macular Pigment—OS	Correlation Coefficient	1.000	0.905 **	−0.714 **	−0.732 **
	Sig. (2-tailed)		0.000	0.000	0.000
	N	57	57	57	57
Macular Pigment—OD	Correlation Coefficient	0.905 **	1.000	−0.682 **	−0.732 **
	Sig. (2-tailed)	0.000		0.000	0.000
	N	57	57	57	57
Screen Time—Weekdays	Correlation Coefficient	−0.714 **	−0.682 **	1.000	0.846 **
	Sig. (2-tailed)	0.000	0.000		0.000
	N	57	57	57	57
Screen Time—Weekend	Correlation Coefficient	−0.732 **	−0.732 **	0.846 **	1.000
	Sig. (2-tailed)	0.000	0.000	0.000	
	N	57	57	57	57

** Correlation is significant at the 0.01 level (2-tailed).

## Data Availability

The original contributions presented in this study are included in the article. Further inquiries can be directed to the corresponding author.

## Abbreviations

The following abbreviations are used in this manuscript:

MPOD	Macular Pigment Optical Density
MP	Macular pigment
L	Lutein
Z	Zeaxanthin
MZ	Meso-zeaxanthin
OD	Oculus dexter
OS	Oculus sinister
HFP	Heterochromatic Flicker Photometry
FFQ	Food frequency questionnaire
